# Bac*Dive* – The Bacterial Diversity Metadatabase in 2016

**DOI:** 10.1093/nar/gkv983

**Published:** 2015-09-30

**Authors:** Carola Söhngen, Adam Podstawka, Boyke Bunk, Dorothea Gleim, Anna Vetcininova, Lorenz Christian Reimer, Christian Ebeling, Cezar Pendarovski, Jörg Overmann

**Affiliations:** Leibniz Institute DSMZ-German Collection of Microorganisms and Cell Cultures, Braunschweig, Germany

## Abstract

Bac*Dive*–the Bacterial Diversity Metadatabase (http://bacdive.dsmz.de) provides strain-linked information about bacterial and archaeal biodiversity. The range of data encompasses taxonomy, morphology, physiology, sampling and concomitant environmental conditions as well as molecular biology. The majority of data is manually annotated and curated. Currently (with release 9/2015), Bac*Dive* covers 53 978 strains. Newly implemented RESTful web services provide instant access to the content in machine-readable XML and JSON format. Besides an overall increase of data content, Bac*Dive* offers new data fields and features, e.g. the search for gene names, plasmids or 16S rRNA in the *advanced search*, as well as improved linkage of entries to external life science web resources.

## INTRODUCTION

Though usually invisible to the human eye, bacteria and archaea are omnipresent in soils, water and our bodies. Nevertheless, in comparison to their great species variety, the information about their individual functions, interaction with higher taxa as well as their importance for a functional ecosystem is still poorly understood. After a bacterial strain is isolated, the subsequent investigations, publications and the deposit in a culture collection result in an increasing amount of information that typically is broadly scattered over many different scientific journals, books, culture collection catalogues and life science databases around the world. Since these data are an important basis for future research in the life sciences it is essential to mobilize, harmonize and match scattered scientific information about microbial strains in order to foster a seamlessly structured database content and provide an easy and reliable access to the data.

Bac*Dive*–the Bacterial Diversity Metadatabase (http://bacdive.dsmz.de) is maintained and curated at the Leibniz Institute DSMZ—German Collection of Microorganisms and Cell Cultures (DSMZ, www.dsmz.de) and was launched in April 2012 (1). Bac*Dive* provides structured information on a wide range of bacterial and archaeal strains, covering their taxonomy, morphology, physiology, cultivation, geographic origin, application, interaction, or INSDC (2) deposited sequences for genomes,16S rRNA and marker gene data. The data in Bac*Dive* are all strain-associated, predominantly manually annotated and linked to the respective annotation references. A small amount of data is recovered by automated text processing only. Data derived by automated procedures are clearly labelled as such. The source material for the annotation includes detailed internal descriptions of culture collections, expert-compiled compendia on strains and, to an increasing extent, relevant primary scientific literature.

Bac*Dive* constantly gained content over the past two years. This was mostly achieved through a continuous annotation activity and an addition of new Bac*Dive* data fields, especially in the sections *molecular biology* and *morphology and physiology*. Other major developments have been the implementation of RESTful web services and the efficient linking of BacDive contents with other relevant life science databases.

## CONTENTS OF BACDIVE

### Bac*Dive* data statistics

Bac*Dive* strives to provide information on all aspects of microbial diversity and therefore is not limited to specific thematic aspects or to certain groups of bacteria or archaea. It covers strain-associated information on taxonomy, morphology, physiology, sampling and concomitant environmental conditions as well as molecular biology and strain availability. All available information for each strain are structured, organized and displayed according to seven sections (Table 1). For each section an average increase of 129% (range, 7–356%) of database content have been recorded since 2013.

**Table 1.** The seven sections of Bac*Dive* together with selected subjects to illustrate the thematic coverage of each section

Section Name	Selected subjects of the section	Entries per section* 2013	Entries per section* 2015	increase in% 2013–2015
Name and taxonomic classification	domain, phylum, class, family, genus, species and subspecies (if the referring taxonomic ranks are already assigned), available the full scientific name and type strain status	23 458	106 952	356%

Morphology and physiology	utilized substrates, known produced compounds, tolerance level towards for several substances/antibiotics, murine types, lysis/decomposition ability	11 521	39 060	239%

Culture and growth conditions	cultivation media compositions, growth temperatures, pH, salt concentrations, fumigation, lightning constrains	21 605	24 252	12%

Isolation, sampling and environmental information	geographic location (to continent, country, city and further details, e.g. sea or region, geographic coordinates), environmental conditions at sampling time, utilized enrichment media	20 769	22 448	8%

Application and interaction	medical, biotechnical or industrial application, strain associated patents, risk group classification, biotic relationship, potential host relations	14 639	15 680	7%

Molecular biology	genotype information (connected to whole genome or 16S rRNA sequencing results, secondary and tertiary sequence analysis), e.g. INSDC sequence accession numbers, sequence length, GC-content, applied analysis methods	14 591	17 921	23%

Strain availability	depository history, holding biological resource center, culture collection identifiers	18 459	66 832	262%

∑ overall entries		125 042	293 145	134%

Given are the numbers of entries per section in the years 2013–2015 and the percentage increase of entries in the referring time period. *Distinct combination of the respective strain, the annotation source reference for the data entry, the annotated data entry/point.

The current Bac*Dive* release (9/2015) covers more than 53 900 strains distributed over more than 2020 genera and 10 247 species. 9094 strains in Bac*Dive* represent type strains of their species.

### New data fields

The relational database behind Bac*Dive* was initially constructed by definition of over 400 potential data fields for the handling of data that cover all aspects of microbial diversity. Not all of these data fields are applicable for each strain and not every field can be filled when the initial data acquisition procedure of a new strain takes place. Nevertheless, with each release we are striving to increase the set of active data fields, derived out of the pool of potential fields. Furthermore, with each content update we check if currently empty fields can be annotated for any of the strains that are already recorded in Bac*Dive*.

**Figure 1.**
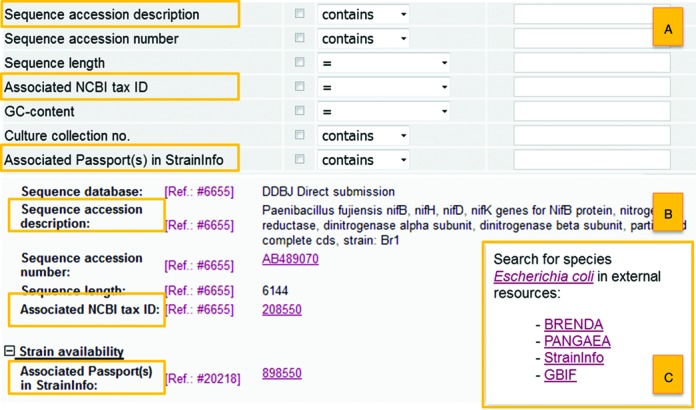
New features in the Bac*Dive* webportal: The *advanced search* (**A**) has been extended by additional search fields *sequence accession description*, *associated NCBI tax ID*, *Associated Passport(s) in StrainInfo*. These additional fields are also shown in the *strain detail view* (**B**). The new feature for facilitated searching in external web resources can also be found in the *strain detail view* (**C**).

In the current release (9/2015), the Bac*Dive* relational database encompasses a total of 233 active data fields, an increase of 54 fields compared to release 9/2013. Some of the recently added active fields provide information that enables better linkage of Bac*Dive* content to other life science data resources and knowledge bases. With respect to other data resources the *strain detail view* of Bac*Dive* has been extended in 2014 by the respective strain passport ID of StrainInfo (3) and by providing a direct link (Figure 1B) to the StrainInfo.net entry where possible. Furthermore, we extended our information on taxonomic classification, which is derived by the taxon reference list of *Prokaryotic Nomenclature up-to-date*. In the *strain detail view* additional information on the status of the species and the higher taxa as well as the respective publication details of the International Journal of Systematic and Evolutionary Microbiology (IJSEM) are routinely updated.

For the current release 9/2015 we massively improved the *molecular biology* section of all strains listed in Bac*Dive* by an increase of content (3330 entries) (Table 1), additional active data fields (Figure 1B) and enhanced search functionalities (Figure 1A). Entries associated to INSDC (2) accession numbers (Figure 1B) are supplemented with a brief description of the corresponding sequence, as well as with the corresponding ID of the NCBI taxonomy (4). This additional information facilitates the re-use of Bac*Dive* derived data as a comprehensive basis for individually defined data analyses.

### A focus on data mobilization for strains of *Actinobacteria*

*Actinobacteria* are ubiquitous in soil and fresh water habitats but can also be found in marine samples. In addition, members of the phylum are of great relevance for medicine, biotechnology or agriculture since they harbour a great variety of pathways for the biosynthesis of secondary compounds, and are capable of producing bioactive metabolites or of degrading pollutants. Several representatives of the *Actinobacteria* are pathogens. Finally, many *Actinobacteria* are multicellular and capable of conspicuous morphological differentiation that involves the formation of different types of mycelia, of sporangia and of exospores. In 2013 and 2014, Bac*Dive* therefore focused on data mobilization for strains of this bacterial phylum.

Mobilization of the unique data sets on *Actinobacteria* was possible by using two particularly rich knowledge sources: (i) the *Compendium of Actinobacteria*, compiled by PD Dr Joachim Wink (https://www.dsmz.de/bacterial-diversity/compendium-of-actinobacteria.html) and (ii) the description of the *Jena Microbial Resource Collection* (JMRC), that is managed jointly by the Leibniz Institute for Natural Product Research and Infection Biology—Hans Knöll Institute (HKI) and the Friedrich Schiller University in Jena.

The *Compendium of Actinobacteria* is a comprehensive electronic PDF manual compiled and constantly amended by PD Dr Joachim Wink for over 15 years. It provides species descriptions, cultivation assays, morphologic characterizations and metabolic profiles. Bac*Dive* focused on the transfer of the available data into structured and systematically searchable database content. As part of this mobilization Bac*Dive* was able to integrate data of the substrate utilization (8151), of assay results for the commercial systems bioMérieux API® kits (2261), of the production of secondary compounds (144), the cultivation media and conditions (1151), inhibitory substance concentrations (597), and morphological descriptions (13 462) derived from the *Compendium of Actinobacteria*. The data set on *Actinobacteria* was supplemented by 820 additional data points that were provided by JMRC and that cover information on natural products, ability of degradation of biomolecules or organic compounds, resistances and related INSDC sequence accessions of the curated strains.

### Pilot study towards the mobilization and annotation of data from scattered sources

Bac*Dive* strives to increase the manually annotated content that is derived from multiple publications and other knowledge sources, and to merge these data with those from collection descriptions and with data on morphological traits directly provided by scientists. In order to determine the amount of data that can be mobilized through literature mining, to develop suitable strategies for the straightforward annotation of published articles at larger scales, and to identify possible pitfalls, we conducted the mobilization of all available data for a test set of five bacterial strains. For this pilot study we focused on members of the phylum *Acidobacteria* since this bacterial group is highly abundant and relevant for nitrogen and carbon cycles in soils. The following strains were chosen: *Aridibacter kavangonensis* Huber *et al*. 2014 strain Ac_23_E3, *Aridibacter famidurans* Huber *et al*. 2014 strain A22_HD_4H (5), *Blastocatella fastidiosa* Fösel *et al*. 2013 strain A2_16 (6), *Edaphobacter aggregans* Koch *et al*. 2008 emend. Dedysh *et al*. 2012 strain WBG 1 and *Edaphobacter modestus* Koch *et al*. 2008 strain JBG-1 (7). An average of 276 (range, 180–319) data points per strain could be mobilized and added to Bac*Dive*. The resulting, highly detailed metabolic, physiological and ecological profile now provides an improved basis for studies of the adaptation, possible ecological functions and potential biotechnological applications of these strains.

## SEARCH FEATURES

The Bac*Dive* portal offers an easy-to-use *simple search* and additional powerful *advanced search* functionalities that allows the combination of more than 35 search fields for text and numerical data. The *advanced search* has been extended by the search fields *sequence accession description*, *associated NCBI tax ID*, *Associated Passport(s) in StrainInfo* (Figure 1A). The query field *sequence accession description* allows for free text searches of gene names, plasmids or filtering by applying the search term ‘rRNA’ or ‘complete genome’.

In the *strain detail view* a feature (Figure 1B) for facilitated and comfortable searching in external web resources was integrated. Direct links trigger queries for searching for the referring species in external web resources, e.g. BRaunschweig ENzyme DAtabase (BRENDA) (8), Global Biodiversity Information Facility (GBIF, http://www.gbif.org), PANGAEA information system (http://www.pangaea.de) and StrainInfo.net (3).

## ACCESSIBILITY

The Bac*Dive* portal is freely accessible via the *simple search* and *advanced search* options. The user may select particular strains and compile a *download selection* that can be exported in a CSV spreadsheet format.

### New RESTful web service

Representational State Transfer (REST) web services provide a simple way to access the data collection without the need for downloading, parsing and preparing an entire database for local queries. The major advantage of these web service interfaces is that they avoid specific parsing routines that otherwise would have to be adjusted each time when text file structures or database organization are changing. Since the launch of the portal in 2012, a web service interface has been the feature most frequently requested by Bac*Dive* users. Therefore, we established two web services (http://bacdive.dsmz.de/api/) for retrieving contents of Bac*Dive* and related information resources in a machine-readable form. Firstly, the Bac*Dive* portal now offers a web service interface for Bac*Dive* content. Secondly, the portal enables accessing the widely used taxon reference list *Prokaryotic Nomenclature Up-To-Date* (PNU, https://www.dsmz.de/bacterial-diversity/prokaryotic-nomenclature-up-to-date.html) via the programming interface provided.

The Bac*Dive* web service (http://bacdive.dsmz.de/api/bacdive/) offers several possibilities to query detailed strain information. It allows the retrieval by genus, species and/or subspecies, a given culture collection number or an INSDC accession number. The response is always listed together with the corresponding Bac*Dive* entry number (a stable numeric identifier for a strain in Bac*Dive*) and is formatted as URL in the web service. Additionally, direct access to strain details is given by usage of the endpoint for a given Bac*Dive* entry number. Currently 99 data fields are reachable via the Bac*Dive* web service. If a data field within BacDive is not filled with content this field is omitted in the web service response. This routine is similar to the setup of the BacDive's *strain detail view* in the portal.

The *Prokaryotic Nomenclature Up-To-Date* web service offers a machine-readable facsimile of the corresponding taxon reference list, which is expert-curated and published at the Leibniz-Institute DSMZ. This list comprises a compilation of all names of Bacteria and Archaea, which have been validly published according to the Bacteriological Code (9) since 1 Jan 1980, as well as all nomenclatural changes, which have been validly published since that date (10). *Prokaryotic Nomenclature Up-To-Date* and its web service back end are routinely updated following the release of each new issue of the IJSEM. In particular, the *Prokaryotic Nomenclature Up-To-Date* web service offers lists of all included species, genera, families and classes and also allows for the retrieval of data by the name of a genus, species and/or subspecies or a given a culture collection number. In addition, species name synonyms can be retrieved under the header *reclassified*, together with the corresponding valid name. The responses of each endpoint contain comprehensive information on the respective taxon, its status, publication details, a list of culture collections strain numbers (culture collection accession numbers) of the type strain, accompanied by links to external web resources, such as the corresponding entries in the List of Prokaryotic names with Standing in Nomenclature (LPSN) (11). The *Prokaryotic Nomenclature Up-To-Date* web service will be one of the external taxon reference sources in the upcoming release of the Global Genome Biodiversity Network (GGBN) data portal (12) and in the near future will be adapted for the Terminology Server (http://terminologies.gfbio.org) of the German Federation for Biological Data (GFBio) (13).

All Bac*Dive* web service resources are free to use. Interested users only need to register for the desired web service in order to gain full access. The web services can be retrieved in machine-readable XML and JSON format. Therefore, they are largely independent of the programming language used or the webserver implementation on the client site. The endpoints, the recommended parameters and syntax are comprehensibly documented in browsable html pages and are accompanied by exemplary queries. These pages provide a quick introduction into Bac*Dive* web services and provide all the details about their use.

The Bac*Dive* web services are available since release 9/2014. They may be enhanced in future releases with additional endpoints or respectively additional fields. During the alpha and beta test period, we already received positive feedback and constructive suggestions for improvement by numerous users and cooperating partner databases. Input and suggestions from our users are highly appreciated.
